# A phase 3 tRial comparing capecitabinE in combination with SorafenIb or pLacebo for treatment of locally advanced or metastatIc HER2-Negative breast CancEr (the RESILIENCE study): study protocol for a randomized controlled trial

**DOI:** 10.1186/1745-6215-14-228

**Published:** 2013-07-22

**Authors:** José Baselga, Frederico Costa, Henry Gomez, Clifford A Hudis, Bernardo Rapoport, Henri Roche, Lee S Schwartzberg, Oana Petrenciuc, Minghua Shan, William J Gradishar

**Affiliations:** 1 Memorial Sloan-Kettering Cancer Center, 1275 York Avenue, New York, NY 10065, USA; 2 Hosp Sirio Libanes, R. Dona Adma Jafet, 91 - Bela Vista, Sao Paulo, Brazil; 3 Instituto Nacional de Enfermedades Neoplasicas, Av Angamos Este 2520, Surquillo, Lima, Peru; 4 The Medical Oncology Centre of Rosebank, Lumley House, 177 Jan Smuts Avenue, Parktown North, Johannesburg 2193, South Africa; 5 Institut Claudius Regaud, 20-24, rue du Pont Saint Pierre 31052, Toulouse, France; 6 West Clinic, 100 North Humphreys Boulevard, Memphis, TN 38120, USA; 7 Bayer HealthCare Pharmaceuticals, PO Box 1000 Montville, NJ 07045, USA; 8 Feinberg School of Medicine, Northwestern University, 676 N St Clair Street, Suite 850, Chicago, IL 60610, USA

**Keywords:** Metastatic breast cancer, Sorafenib, Capecitabine

## Abstract

**Background:**

Sorafenib is an oral multikinase inhibitor with antiangiogenic/antiproliferative activity. A randomized phase 2b screening trial in human epidermal growth factor receptor 2 (HER2)-negative advanced breast cancer demonstrated a significant improvement in progression-free survival (PFS) when sorafenib was added to capecitabine versus placebo (median 6.4 versus 4.1 months; hazard ratio = 0.58; *P* = 0.001). Most drug-related adverse events were Grade 1/2 in severity with the exception of Grade 3 hand-foot skin reaction/syndrome (44% versus 14%, respectively). These results suggest a role for the combination of sorafenib and capecitabine in breast cancer and supported a phase 3 confirmatory trial. Here we describe RESILIENCE - a multinational, double-blind, randomized, placebo-controlled, phase 3 trial - assessing the addition of sorafenib to first- or second-line capecitabine in advanced HER2-negative breast cancer.

**Methods/design:**

Eligibility criteria include ≥18 years of age, ≤1 prior chemotherapy regimen for metastatic disease, and resistant to/failed taxane and anthracycline or no indication for further anthracycline. Prior treatment with a vascular endothelial growth factor inhibitor is not allowed. Patients with significant cardiovascular disease or active brain metastases are not eligible. Patients are stratified by hormone-receptor status, geographic region, and prior metastatic chemotherapy status and randomized (1:1) to capecitabine (1000 mg/m^2^ orally twice daily (BID), days 1 to 14 of 21) in combination with sorafenib (orally BID, days 1 to 21, total dose 600 mg/day) or matching placebo. Capecitabine and sorafenib/placebo doses can be escalated to 1250 mg/m^2^ BID and 400 mg BID, respectively, as tolerated, or reduced to manage toxicity. Dose re-escalation after a reduction is allowed for sorafenib/placebo but not for capecitabine. This dosing algorithm was designed to mitigate dermatologic and other toxicity, in addition to detailed guidelines for prophylactic and symptomatic treatment. Radiographic assessment is every 6 weeks for 36 weeks, and every 9 weeks thereafter. The primary endpoint is PFS by blinded independent central review (Response Evaluation Criteria in Solid Tumors 1.1 criteria). Secondary endpoints include overall survival, time to progression, overall response rate, duration of response, and safety. Enrollment began in November 2010 with a target of approximately 519 patients.

**Discussion:**

RESILIENCE will provide definitive PFS data for the combination of sorafenib and capecitabine in advanced HER2-negative breast cancer and better characterize the benefit-to-risk profile.

**Trial registration:**

Clinicaltrials.gov, NCT01234337

## Background

In recent decades, advances in the treatment of metastatic breast cancer (MBC) have provided incremental improvement in disease control and survival [1]. Novel chemotherapies (for example, paclitaxel, capecitabine) for MBC and the introduction of hormone and other targeted therapies (for example, trastuzumab) have corresponded to a steady increase in survival. Despite these improvements, the primary goal of treatment remains palliative with about half of patients succumbing to the disease within 3 years [1-4].

Breast cancer is a heterogenous disease with systemic treatment options primarily based on hormone receptor and human epidermal growth factor receptor 2 (HER2) status, but also emerging therapies that target other molecular pathways involved in development, progression, resistance, and metastasis, such as antiangiogenics and poly(adenosine diphosphate ribose) polymerase (PARP) inhibitors [5]. While management of early disease stages is well defined, management of MBC, particularly HER2-negative disease, is complex as standard treatment strategies are not well established [6,7]. The optimal sequence or combination of agents has not been standardized and is dependent on a variety on intra-patient factors. Combination chemotherapy regimens have been shown to provide clinical benefit in MBC but are associated with significant toxicities and have not shown an overall survival (OS) benefit compared with sequential use of agents [3,8,9]. Novel combinations are needed that improve survival, disease control, and quality of life (QoL) with acceptable toxicity.

Although single-agent use of antiangiogenics has demonstrated only modest efficacy in MBC [10-14], studies in HER2-negative MBC have demonstrated the clinical benefit of adding antiangiogenics to chemotherapy. This was first established with bevacizumab, a monoclonal antibody targeting vascular endothelial growth factor (VEGF), a potent angiogenic factor [15-19]. In the E2100 phase 3 study, the addition of bevacizumab to first-line paclitaxel demonstrated a clinically significant improvement in progression-free survival (PFS) for patients with HER2-negative MBC [16]. PFS increased from 5.9 months for paclitaxel alone to 11.8 months for bevacizumab plus paclitaxel (hazard ratio [HR] = 0.60; 95% confidence interval [CI] = 0.51 to 0.70; *P* < 0.001). The combination was tolerable, although some serious events occurred infrequently. Subsequent studies of bevacizumab in combination with selected chemotherapies have also demonstrated significant improvement in PFS, but the benefit has been notably more modest than the E2100 results [15,18,19]. Furthermore, an OS benefit has not been demonstrated across these bevacizumab studies.

A number of factors might explain the discrepancy between the PFS benefit and the lack of improvement in OS. It is difficult to demonstrate an OS benefit in first-line MBC because treatments after progression likely confound data [20]. In addition, preclinical data suggest that antiangiogenic treatment may result in more aggressive disease at the time of progression, possibly through increased invasiveness of tumor cells and/or by switching to alternative angiogenic pathways to re-establish tumor vascularization [21,22]. This led clinicians to investigate whether other antiangiogenic agents that have a broader spectrum of activity than bevacizumab, such as the tyrosine kinase inhibitors sunitinib and sorafenib, might provide benefit in HER2-negative MBC. While phase 3 studies with sunitinib have been disappointing thus far [10,23-25], a series of phase 2b randomized, placebo-controlled trials have collectively demonstrated sorafenib activity when combined with selected chemotherapies [26-29].

Sorafenib, an oral multikinase inhibitor with both antiproliferative and antiangiogenic activity, is indicated for advanced renal cell and hepatocellular carcinomas [30,31]. Sorafenib targets VEGF receptors-1, -2, and −3, platelet-derived growth factor receptor β, Raf kinase, c-KIT, and Flt-3 [32]. Preclinical data in breast tumor models suggest that adding sorafenib to cytotoxic agents may provide synergistic/additive antitumor effects and may help overcome resistance to cytotoxic agents [33-35].

The Trials to Investigate the Efficacy of Sorafenib (TIES) in Breast Cancer Program was developed to rapidly assess the efficacy and safety of sorafenib in combination with selected systemic therapies for HER2-negative MBC, to determine if phase 3 confirmatory trials should be pursued, and to inform the design of these trials. The TIES program was developed by independent investigators with support from industry to collectively identify settings in HER2-negative MBC where the addition of sorafenib might be of benefit. The program consists of four double-blind, randomized, placebo-controlled, phase 2b screening trials in patients with HER2-negative advanced breast cancer that assessed sorafenib in combination with first- or second-line capecitabine (SOLTI-0701) [26], first-line paclitaxel (NU07B1) [28], first- or second-line gemcitabine or capecitabine (AC01B07) in patients who had progressed during or after a regimen containing bevacizumab [27], and first-line docetaxel and/or letrozole (FM-B07-01) [29]. Two of the TIES programs indicated a potential role for sorafenib in this patient population when used in combination with selected chemotherapies [26,27]. More specifically, results of the SOLTI-0701 supported a phase 3 confirmatory study of the sorafenib-capecitabine combination [26]. AC01B07 also met its primary endpoint, but the PFS benefit with the sorafenib combination was too modest to support a phase 3 trial of similar design [27]. NU07B1 did not demonstrate a statistically significant benefit in PFS with the sorafenib combination but showed a statistically significant improvement in time to progression (TTP) [28]. There was no clinical benefit associated with the sorafenib combination in the FM-B07-01 study [29].

During SOLTI-0701, the addition of sorafenib to first- or second-line capecitabine significantly improved the primary endpoint of PFS compared with placebo (median 6.4 versus 4.1 months; HR = 0.58; 95% CI = 0.41 to 0.81; *P* = 0.001) and the secondary endpoint of TTP (median 6.8 versus 4.1 months; HR = 0.56; 95% CI = 0.39 to 0.80; *P* = 0.001) [26]. There was no significant improvement in overall survival (median 22.2 versus 20.9 months; HR = 0.86; 95% CI = 0.61 to 1.23; *P* = 0.42).

Generally, the combination of sorafenib and capecitabine was manageable, but dose reductions were more common than in the placebo arm [26]. During SOLTI-0701, the starting dose was 1000 mg/m^2^ twice daily (BID) capecitabine (first 14 days of a 21-day cycle) and 400 mg BID (continuously) for sorafenib/placebo. Dose interruptions and reductions were allowed to manage toxicity. Dose reductions in the sorafenib arm were 53% for sorafenib and 78% for capecitabine compared with 14% for placebo and 33% for capecitabine in the placebo arm. The average daily dose of study drugs was lower in the sorafenib arm (mean 584 mg sorafenib and 1461 mg/m^2^ capecitabine) compared with the placebo arm (mean 745 mg placebo and 1839 mg/m^2^ capecitabine). Most patients remained on treatment until disease progression in the sorafenib and placebo arms (63% and 82%), but some discontinued treatment due to adverse events (AEs) (20% versus 9%, respectively). The average duration of treatment in the sorafenib arm was 33.8 weeks for sorafenib and 33.1 weeks for capecitabine, with corresponding values of 22.5 and 22.2 weeks in the placebo arm. The most common toxicity was hand-foot skin reaction (HFSR) associated with sorafenib and hand-foot syndrome (HFS) associated with capecitabine, which occurred more frequently in the sorafenib arm than the placebo arm (90% versus 66% for any grade and 44% versus 14% for Grade 3) and occurred earlier (median time to onset 14 versus 64 days, respectively, for any grade). Other Grade 3/4 events were comparable between the groups.

The results of SOLTI-0701 supported the rationale for a similar confirmatory trial in the phase 3 setting, but also indicated that the study design would require amendments to the dosing schema to address the incidence of HFSR/HFS in addition to more aggressive prevention and management strategies. Here we present the study design of the phase 3 RESILIENCE trial (tRial comparing capecitabinE in combination with SorafenIb or pLacebo for treatment of locally advanced or metastatIc HER2-Negative breast CancEr).

## Methods/design

RESILIENCE is a multinational, randomized, double-blind, placebo-controlled, phase 3 trial designed to assess the efficacy and safety of sorafenib in combination with capecitabine in patients with locally advanced or metastatic HER2-negative breast cancer who received previous treatment with a taxane and anthracycline. The study is being conducted in accordance with Good Clinical Practice guidelines, the guiding principles of the Declaration of Helsinki, and applicable local laws and regulations. The study protocol has been approved by both Regulatory Authorities and internal review boards of the participating centers. All patients will provide written informed consent. The trial is registered at ClinicalTrials.gov (NCT01234337). (For a complete listing of study centers see Additional file 1).

### Patient population and study design

**Figure 1:**
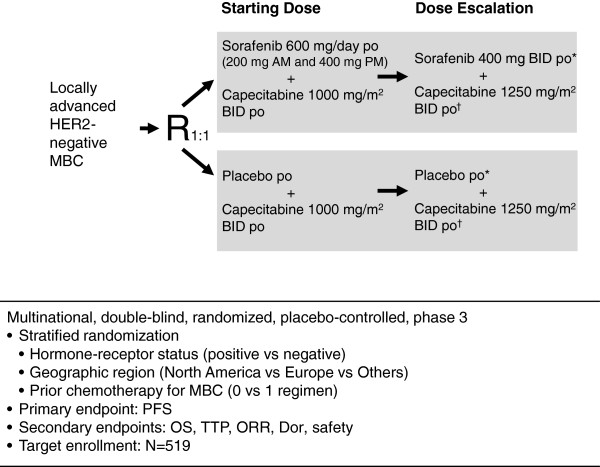
**Study design and treatment schema of RESILIENCE.** *If treatment is well tolerated after Cycle 1; ^†^if sorafenib/placebo 400 mg BID is well tolerated. BID, twice daily; DoR, duration of response; MBC, metastatic breast cancer; ORR, overall response rate; OS, overall survival; PFS, progression-free survival; po, oral; R, randomization; RESILIENCE, tRial comparing capecitabinE in combination with SorafenIb or pLacebo for treatment of locally advanced or metastatIc HER2-Negative breast CancEr; TTP, time to progression.

Patients at least 18 years of age with HER2-negative advanced breast cancer who have been treated with a maximum of one prior chemotherapy regimen for metastatic disease are eligible to enroll. Prior treatment with both a taxane and an anthracycline is required, but prior treatment with a VEGF inhibitor (for example, bevacizumab) is not allowed. Table 1 summarizes major eligibility criteria. Patients will be stratified by hormone-receptor status (estrogen and/or progesterone receptor positive versus both negative), geographic region (North America versus Europe versus Other), and number of prior MBC chemotherapy regimens (0 or 1), then randomized (1:1) to receive either sorafenib or placebo in combination with capecitabine (Figure 1).

**Table 1:** Eligibility criteria for RESILIENCE

**Key inclusion criteria**	**Key exclusion criteria**
● Age ≥18 years	● HER2-positive disease
● Life expectancy ≥12 weeks	● Unknown estrogen and progesterone receptor status
● Histologically or cytologically confirmed HER2-negative breast adenocarcinoma	● Previous treatment with a VEGF inhibitor
● Locally advanced (non-resectable) or metastatic disease	● Symptomatic brain metastases
● Measurable or clinically evaluable disease according to Response Evaluation Criteria in Solid Tumors (RECIST 1.1)	● Recent (<4 weeks before entry) major surgery, open biopsy, or significant traumatic injury
● Resistant to or failed prior taxane and an anthracycline or further anthracycline therapy is not indicated	● Uncontrolled hypertension, or active or clinically significant cardiac disease
● No more than one previous chemotherapy regimen for metastatic disease	● Thrombotic, embolic, venous, or arterial events, or other bleeding disorders within the past 6 months
● Prior adjuvant or neoadjuvant chemotherapy allowed	● Any hemorrhage/bleeding event of Grade 3 or above within past 4 weeks
● Prior hormonal therapy for locally advanced or metastatic disease allowed	
● Prior chemotherapy, radiation, or hormonal therapy discontinued ≥4 weeks before randomization; previously irradiated areas must not be the only site of disease	
● ECOG Performance Status of 0 or 1	
● Adequate hepatic and renal function	

*ECOG* Eastern Cooperative Oncology Group, *HER2* human epidermal growth factor receptor 2, *RESILIENCE* tRial comparing capecitabinE in combination with SorafenIb or pLacebo for treatment of locally advanced or metastatIc HER2-Negative breast CancEr, *VEGF* vascular endothelial growth factor.

Patients will receive sorafenib or matching placebo BID on a continuous basis. The initial daily dose of sorafenib is 600 mg taken as one 200-mg tablet in the morning and two 200-mg tablets in the evening. The 600-mg dose was selected because it corresponds to the average daily dose (mean 584 mg/day) in the sorafenib arm of the SOLTI-0701 study [26]. Capecitabine is administered orally on days 1 through 14 of a 21-day cycle at a starting dose of 1000 mg/m^2^ BID. Studies have established the efficacy and safety of capecitabine 1000 mg/m^2^ BID [36]. The daily dose of sorafenib or matching placebo may be increased to 400 mg BID if the 600-mg dose is well tolerated (fatigue, dermatologic toxicities, or gastrointestinal toxicities are no greater than Grade 1) after a 21-day cycle. If the 400-mg BID dose of sorafenib/placebo is well tolerated in a 21-day cycle, then the capecitabine dose may be increased to 1250 mg/m^2^ BID in the subsequent cycle.

### Management of adverse events

Dose reductions, interruptions, and discontinuation of study drugs are required to manage toxicity and are detailed in the study protocol by toxicity type and grade, which also specifies how dosing of the study drug(s) should be modified. Re-escalation of sorafenib/placebo after a dose reduction is permitted per protocol guidance, but dose re-escalation is not permitted for capecitabine as per the capecitabine label. If an AE leads to dose interruption of more than 21 days, recurs too frequently and leads to dose reductions, and/or is too severe (for example, Grade 4 non-hematologic toxicities), one or both study drugs may be permanently discontinued. Sorafenib treatment cannot be continued if capecitabine has been discontinued permanently, but capecitabine may continue if sorafenib has been permanently discontinued. Proactive management of HFSR/HFS leads to better management of these toxicities, allowing patients to remain on treatment. Based on the experience in the SOLTI-0701 study, the RESILIENCE protocol includes comprehensive guidelines for prophylactic management (for example, pedicure and moisturizing creams) and symptomatic treatment (for example, topical exfoliants and analgesics) of HFSR/HFS (Table 2), in addition to dose modifications of study drugs.

**Table 2:** Recommended prevention/management strategies for skin toxicities consistent with hand-foot skin reaction/hand-foot syndrome

**Toxicity grade**	**Practical prevention/management strategies for HFSR/HFS**
Grade 0 (preventive strategies)	● Maintain frequent contact with trial physician to ensure early diagnosis of HFSR
	● Practical prevention strategies
	○ Pedicure by a podiatrist for subjects with pre-existing hyperkeratosis
	○ Subjects should avoid hot water, and clothing or activities that can cause friction on the skin
	○ Moisturizing cream should be applied sparingly
	● Padded gloves and open shoes with padded soles should be worn to relieve pressure points
Grade 1 any occurrence	● Continue preventive strategies and in addition:
	○ Soak hands in cool water
	○ Apply petroleum jelly to moist skin
	● In the case of hyperkeratotic lesions, exfoliate the hands or feet and apply moisturizing cream immediately afterwards
Grade 2 or 3 any occurrence	● Continue supportive/management measures and add analgesic(s) for pain
	● Dose modifications to study treatments per protocol guidance

*HFS:* hand-foot syndrome, *HFSR:* hand-foot skin reaction.

### Assessments

Electrocardiogram, blood tests, patient QoL questionnaires, a complete physical examination, and assessment of Eastern Cooperative Oncology Group (ECOG) performance status will be performed at the beginning of each treatment cycle. On a weekly basis during the first 6 weeks of treatment, patients will undergo a brief physical examination comprising assessment of vital signs and a thorough dermatologic examination for HFSR/HFS and other dermatologic toxicities.

Tumor measurements during the study will be conducted using computed tomography scan and/or magnetic resonance imaging, with a bone scan if clinically indicated at the time of screening, on day 1 of Cycle 1 and every 6 weeks from the day of randomization through week 36, and then every 9 weeks thereafter. Treatment response will be evaluated using Response Evaluation Criteria in Solid Tumors (RECIST 1.1) criteria [37].

### Study endpoints

#### Primary and secondary endpoints

The primary efficacy endpoint is PFS by blinded independent central review. Secondary efficacy endpoints are OS, TTP, overall response rate (ORR), and duration of response (DoR). Safety evaluations comprise AE reporting by standard methods (National Cancer Institute-Common Terminology Criteria for Adverse Events v4.0) and assessment of laboratory abnormalities.

#### Patient-reported outcomes

Patient-reported outcomes (PROs) include an evaluation of breast cancer symptoms using the Functional Assessment of Cancer Therapy-Breast Symptom Index (8 items) (FBSI-8 questionnaire) [38,39] and health-related quality of life (HRQoL) using the EuroQoL 5-Dimension Questionnaire (EQ-5D) [40].

#### Pharmacokinetics

Pharmacokinetic analysis of capecitabine, 5-fluorouracil (5-FU) (the active metabolite of capecitabine), and sorafenib exposures will be conducted. Five blood samples each will be collected from approximately 200 patients for these analyses. On day 14 of Cycle 2, samples will be obtained prior to and at 0.5, 1, 2, and 4 hours after the capecitabine dose to estimate capecitabine and 5-FU exposure. Sorafenib exposure will be estimated from a single blood sample on day 14 of Cycle 2.

#### Exploratory biomarkers

Exploratory analyses of cancer biomarkers such as PIK3CA, BRAF, and KRAS will be performed to possibly identify subpopulations of patients with differential response to sorafenib treatment. Plasma samples for biomarker analysis (genetic and non-genetic) will be collected at screening, prior to drug administration in Cycles 1 through 3, and at the end of treatment. Permission to obtain an archived biopsy sample from diagnosis or later will be sought from all patients during screening.

### Statistical analyses

Assuming a 1-sided alpha of 0.005, a power of 98.9%, and a randomization ratio of 1:1 between the sorafenib and placebo groups, 363 events are required to detect a 66.7% increase in PFS. Assuming enrollment at a rate of 20 patients per month, an enrollment ramp-up time of 4 months, a dropout rate of 10%, an exponentially distributed event time, 4.5-month median PFS for the control group, and a 28.4-month enrollment period, a sample size of 519 is estimated with an expected trial duration of 31.4 months.

Efficacy analyses will be performed with the intent-to-treat population. The primary analysis of the study will be performed after approximately 363 PFS events. Analysis of PFS will be performed using a log-rank test, stratified by randomization factors (that is, hormone-receptor status, geographic region, and prior chemotherapy for MBC) with 1-sided alpha of 0.005. All secondary time-to-event endpoints (OS, TTP and DoR) will be analyzed in a similar manner but with a 2-sided alpha of 0.05.

Assuming a median OS of 12 months for the control group, approximately 270 deaths would be expected at the time of the primary analysis of PFS, at which time an interim analysis of OS is planned. For the final analysis of OS, 405 deaths are projected to occur by approximately 47.9 months after the first patient is randomized. Analysis of 405 events will provide 82% power to detect a 33.3% increase in survival with sorafenib compared with placebo with a 2-sided alpha of 0.05, assuming one interim analysis of OS at the time of the PFS analysis. The interim and final analyses of OS will be performed using an O’Brien-Fleming type alpha spending function.

For other endpoints, a Cochran-Mantel-Haenszel test with the randomization factors as strata will be used to compare ORR between treatment arms. PROs will be analyzed by time-adjusted area under the curve with the comparison between treatment arms made using a covariance analysis, if appropriate. The predictive value of baseline biomarker levels (genetic and non-genetic) for the treatment effect in PFS and OS will be assessed using appropriate statistics to determine whether any of the known biological targets of sorafenib are important in assessing efficacy. In addition, correlations between change in biomarker levels from baseline and PFS and OS will be investigated.

## Discussion

Data from the SOLTI-0701 showed a statistically significant improvement in PFS when sorafenib was added to capecitabine as a first- or second-line treatment and supported and informed the development of the phase 3 RESILIENCE study [26]. The RESILIENCE study is similar in design to SOLTI-0701 but with some differences in total daily dose of sorafenib. RESILIENCE will provide definitive PFS data for the addition of sorafenib to capecitabine in patients with advanced breast cancer. It will better characterize the benefit-to-risk profile for these doses of sorafenib and capecitabine and provide information on the benefits of prophylactic management and treatment of HFSR/HFS. Because of the high incidence of HFSR/HFS during SOLTI-0701 as well as the sorafenib mean dose of 584 mg, the RESILIENCE study will start sorafenib at a 600 mg/day (200 mg in the morning and 400 mg in the evening) dose with ability to titrate the dose up to 400 mg BID as tolerated or down to manage toxicity. For SOLTI-0701, the dosing schema started at the highest sorafenib dose of 400 mg BID with the option of titrating down only to manage toxicity. It is of clinical interest to determine if the modifications to the dosing schema from SOLTI-0701 to RESILIENCE, in addition to more aggressive prophylactic and symptomatic treatment guidance, mitigate the incidence, duration, and severity of HFSR/HFS as well as influence the duration of treatment for the sorafenib arm.

The RESILIENCE study will also provide more robust OS data. The SOLTI-0701 study failed to show a difference between treatment arms for OS, but these were phase 2b studies that were not powered for such an analysis [26]. Of interest, a trend of improved survival was observed in the first-line treatment subgroup of the SOLTI-0701 study (HR = 0.67; 95% CI = 0.40 to 1.11) but not in the second-line treatment subgroup (HR = 1.08; 95% CI = 0.65 to 1.78).

The PROs of RESILIENCE will help to better define the benefit-to-risk profile of the sorafenib-capecitabine combination. Maintaining patient QoL has become an important clinical goal of MBC treatment. Although toxicities associated with sorafenib, like HFSR/HFS, are reversible and non-life threatening in most cases, they can have a significant impact on QoL [41]. In addition, the exploratory analysis of biomarkers may help to better characterize the effect of disease biology on treatment response that may be useful in the design of future studies, particularly those that investigate the impact of patient selection on clinical outcomes.

The RESILIENCE study is an essential step for the development of sorafenib in MBC, as it will provide definitive PFS data and more definitive OS data and inform on the management of HFSR/HFS. An effective all-oral combination regimen for HER2-negative disease would be an important addition to the therapeutic armamentarium in this patient population [42].

## Trial status

The RESILIENCE study began enrolling patients in November 2010. The study is being conducted in more than 20 countries and 200 investigational sites.

## Abbreviations

5-FU: 5-fluorouracil; AE: Adverse event; BID: Twice daily; CI: Confidence interval; DoR: Duration of response; ECOG: Eastern Cooperative Oncology Group; HER2: Human epidermal growth factor 2; HFS: Hand-foot syndrome; HFSR: Hand-foot skin reaction; HR: Hazard ratio; HRQoL: Health-related quality of life; MBC: Metastatic breast cancer; ORR: Overall response rate; OS: Overall survival; PARP: Poly(adenosine diphosphate ribose) polymerase; PFS: Progression-free survival; PRO: Patient reported outcome; QoL: Quality of life; RECIST: Response Evaluation Criteria in Solid Tumors; RESILIENCE: TRial comparing capecitabinE in combination with SorafenIb or pLacebo for treatment of locally advanced or metastatIc HER2-Negative breast CancEr; TIES: Trials to Investigate the Efficacy of Sorafenib; TTP: Time to progression; VEGF: Vascular endothelial growth factor

## Competing interests

JB states that he has Scientific Advisory Board positions with both Roche and Bayer-Onyx. FC, HG, CAH, BR, HR, and LSS state that they have no conflicts of interest to declare. OP and MS state that they are employed with and hold stock in Bayer Healthcare US, NJ. WJG states research funding from Bayer-Onyx. All authors state that they have no non-financial competing interests.

## Authors’ contributions

JB contributed to the conception and design of the RESILIENCE study and was involved in drafting and critically revising the manuscript for important intellectual content. WJG, HR, CAH and BR contributed to the conception and design of the RESILIENCE study and were involved in critically revising the manuscript for important intellectual content. FC, HG, LSS and OP were involved in critically revising the manuscript for important intellectual content. MS was involved in critically revising the manuscript for important intellectual content (particularly statistical matters). All authors have given final approval of the version to be published.

## Authors’ information

Dr Baselga’s career is focused on the development of novel molecular targeted agents, with special emphasis in breast cancer. He has directed the pre-clinical and early clinical development of therapies against the epidermal growth factor receptor and the closely related HER2 receptor. His current work has expanded towards the early clinical development of mTOR and PI3Kinase inhibitors. In the process, he has been deeply involved in the creation of a new paradigm for translational and clinical research, generating hypotheses in the laboratory and moving them swiftly into clinical trials. On the administration front, he has led the transformation of the Vall d’Hebron Hospital Oncology from a small clinical service to a full cancer center with a large multidisciplinary research program and currently among the largest in Europe. He accepted a position as Associate Director of the Massachusetts General Hospital Cancer Center (MGHCC) and, in this role, he is implementing infrastructure for the expansion and improvement of the Phase I program. This includes novel clinical trial infrastructure, core facilities focusing on biomarker identification in refractory tumors, and one which carries out “one-thousand cancer cell line” drug screens, and the creation of a new Termeer Center, a state of the art “complex clinical trials unit” at MGH CC.

## Supplementary Material

Additional file 1 RESILIENCE Study Centers by Country. Click here for file
